# Implementation and Access to Pre-exposure Prophylaxis for Human Immunodeficiency Virus by Men Who Have Sex With Men in Europe

**DOI:** 10.3389/fmed.2021.722247

**Published:** 2021-08-25

**Authors:** Bruno Sepodes, João Rocha, Jorge Batista, Maria-Eduardo Figueira, František Dráfi, Carla Torre

**Affiliations:** ^1^Faculdade de Farmácia da Universidade de Lisboa, Department of Pharmacy, Pharmacology and Health Technologies, Lisbon, Portugal; ^2^Laboratory of Systems Integration Pharmacology, Clinical & Regulatory Science–Research Institute for Medicines of the University of Lisbon (iMED.ULisboa), Lisbon, Portugal; ^3^Unidade de Saúde Pública Internacional e Bioestatística, Instituto de Higiene e Medicina Tropical, Universidade NOVA de Lisboa, Lisbon, Portugal; ^4^Faculdade de Farmácia da Universidade de Lisboa, Department of Pharmaceutical Sciences and Medicines, Lisbon, Portugal; ^5^Institute of Experimental Pharmacology and Toxicology, Centre of Experimental Medicine of the Slovak Academy of Sciences, Bratislava, Slovakia

**Keywords:** acquired immunodeficiency syndrome, aids, HIV infection, implementation, pre-exposure prophylaxis, men who have sex with men, Europe, PrEP

## Abstract

Pre-exposure prophylaxis (PrEP) is a significant public health intervention with proven efficacy and safety in the prevention of human immunodeficiency virus (HIV) infection, which has taken a considerable amount of time to reach Europe in relation to their transatlantic counterparts, namely, the United States of America (USA). There, it is perceived as being an essential prevention tool to be integrated within existing medical, behavioral and structural interventions in place for the management and containment of HIV infection in men who have sex with men (MSM). In a region such as Europe, with approximately double the USA population, it is estimated that not even 10% have proper access to PrEP, and given the lack of coordination with healthcare, taking PrEP has to be at their own expense. Here, we identify the reasons behind the 4-year lag in the approval of PrEP in the European Union/European Economic Area (and Europe in general) and explore the efficacy and effectiveness of PrEP needed to be confirmed with some implementation or demonstration studies conducted in the region. Independent of the data gathered, access of MSM to PrEP is far from ideal in Europe and much still needs to be done. The demonstration of the cost-effectiveness of PrEP alongside other social and behavioral factors needs to be addressed, while the clear populations within MSM that will benefit from this intervention are properly identified and make use of the latest recommendations of the World Health Organization that consider not only daily PrEP but also event-driven PrEP. The momentum for the proper implementation of PrEP in the EU is not lost, and with the existence of generics and even new formulations, there is a renewed opportunity for unleashing the public health benefits arising from this pharmacological tool with other interventions in place (e.g., condoms, testing, and counseling).

## Introduction

The 5th of June 2021 marked the sad 40th anniversary of the medical description of the first cases of acquired immune-deficiency syndrome (AIDS), back in 1981 in Los Angeles in the United States of America (USA). Today, many people living in the developed parts of the world believe that the human immunodeficiency virus (HIV) is a danger from the past, a problem that is now resolved (1), though ~2 million people were infected with HIV in 2014 (2) and at the end of 2019, it was estimated that 38 million people were living with HIV (3). In the wise words of Fauci and Lane, “*the dramatic saga of AIDS features an early sense of helplessness and frustration in the face of a mysterious new disease, courage by the afflicted, and the gradual accrual of groundbreaking scientific advances that have brought hope to a formerly desperate situation”* (4). It is undeniable that the last 30 years were associated with progress, given the advancements in science and public health, even if an effective vaccine or cure has not yet been found (1).

Strong global political and financial support enabled global efforts to fight HIV infection (5). The international community is making efforts to commit to the Sustainable Development Goal (SDG) of ending the HIV/AIDS epidemic by 2030, leaving no one behind (6).

To achieve this goal, the joint United Nations Program on HIV/AIDS (UNAIDS) recommended that 3 million people had access to pre-exposure prophylaxis (PrEP) by 2020 (7). In fact, in the last 3 decades the clinical management of HIV became very similar to the management of other chronic diseases such as diabetes and hypertension. Most interestingly, effective treatment of HIV patients has proven to eliminate the risk of HIV transmission to sexual partners, while some highly effective new prevention methods have emerged such as needle-exchange programs and PrEP (1). These achievements are proof that investing in the right programs for the right target populations can change the course of the HIV pandemic (5). This does not mean that current HIV prevention tools are simple to implement. In fact, it is agreed that HIV prevention requires a multifactorial approach encompassing behavioral, structural, and biomedical strategies (5).

Of note, there are global and regional targets established for ending AIDS. In summary, the targets of the SDGs by 2030 aim for zero new infections (90% reduction), zero AIDS deaths (90% reduction) and zero discrimination (8); the Fast-Track targets by 2020 included the reduction of new HIV infections and AIDS-related deaths both to fewer than 500,000 by 2020 at a global scale, and to eliminate HIV stigma and discrimination (8). Importantly, the specific targets set for Europe by 2020 included the incidence reduction of 75% in infections (2010 baseline) and the use of PrEP (without any specific target being mentioned), along with the alignment with the 90–90–90 target (8). Also, for Europe by 2020, no mortality targets were clearly defined, and although elimination of stigma remained a firm objective, this is currently not measured in the EU/EEA space (8).

PrEP is a prevention tool that consists in using antiretrovirals before, during, and after periods of possible sexual exposure to HIV (9), and this use of antiretroviral medications by HIV-uninfected individuals is expected to block HIV acquisition (2). PrEP may be delivered orally or topically, and efforts have recently been made to develop forms of enhanced topical or systemic delivery, namely slow-releasing and long-acting forms, such as vaginal rings or subcutaneous depot (10). This could be of interest in some target groups where optimization of delivery approaches is still needed (2). With this goal, in 2012, the World Health Organization (WHO) advocated the use of tenofovir disoproxil fumarate (TDF) among serodiscordant couples and men who have sex with men (MSM) (2, 11). Two years later, these same suggestions were incorporated into updated HIV clinical management guidelines, “*including a strong recommendation for offering PrEP as a prevention option for MSM”* (2, 12).

Fonner et al. reviewed the effect of oral PrEP containing TDF in 15 randomized clinical trials and three observational studies concluding this is an effective tool to reduce the risk of HIV acquisition across different sexual exposures, different sexes, different PrEP regimens, and even different dosing schemes (2). Besides the use of TDF for PrEP, the combination of TDF and emtricitabine (FTC) was also adopted as an acceptable regimen with comparable effectiveness (2). According to these authors, “*the use of TDF PrEP in the heterosexual populations may be attractive because of its comparable effectiveness, lower cost, greater availability, and lower risk of drug resistance”* (2), and although only one safety study was conducted with TDF PrEP among MSM, safety information was already available from other trials in MSM conducted using FTC/TDF PrEP.

PrEP uptake and adherence among those at higher risk for HIV infection are key determinants of the impact of this strategy. The review performed by Fonner et al. (2), and the review on PrEP acceptability by Koechlin et al. (13), along with cost/feasibly considerations, led the WHO to expand the 2014 recommendation in order to include the support for PrEP to all populations at substantial HIV risk (14).

Despite the recognized role of PrEP as a highly effective prevention tool, the uptake is very different across the world. Here we will focus on the reality of Europe and how PrEP is being used by MSM in this region, compared to other regions of the world, namely in the USA where the uptake of PrEP appears to be more cultured. For completeness, it must be understood that Europe includes the European Union (EU)/European Economic Area (EEA), and the United Kingdom (UK, which until very recently was included in the EU/EEA and is, therefore, a relevant part of the PrEP odyssey in Europe). Of note, literature appears to be scarce regarding completed or ongoing studies in Europe concerning PrEP for MSM, and the same is true for published data regarding access and implementation of PrEP by MSM in Europe.

## Men Who Have Sex With Men as A Key Population in the European Union HIV Epidemic

In 2019, there was a reduction of 5.2% in new HIV diagnosis in relation to the previous year (15, 16). As in the previous year, in 2019, sex between men remained the most commonly reported route of HIV transmission (50.6%) among those for whom route of transmission was known and accounted for over 38.7% of new HIV diagnoses in the region and for more than 60% of new HIV diagnosis in 10 countries of the region: Croatia, Czech Republic, Germany, Hungary, Iceland, the Netherlands, Poland, Slovakia, Slovenia, and Spain (16).

**Figure 1:**
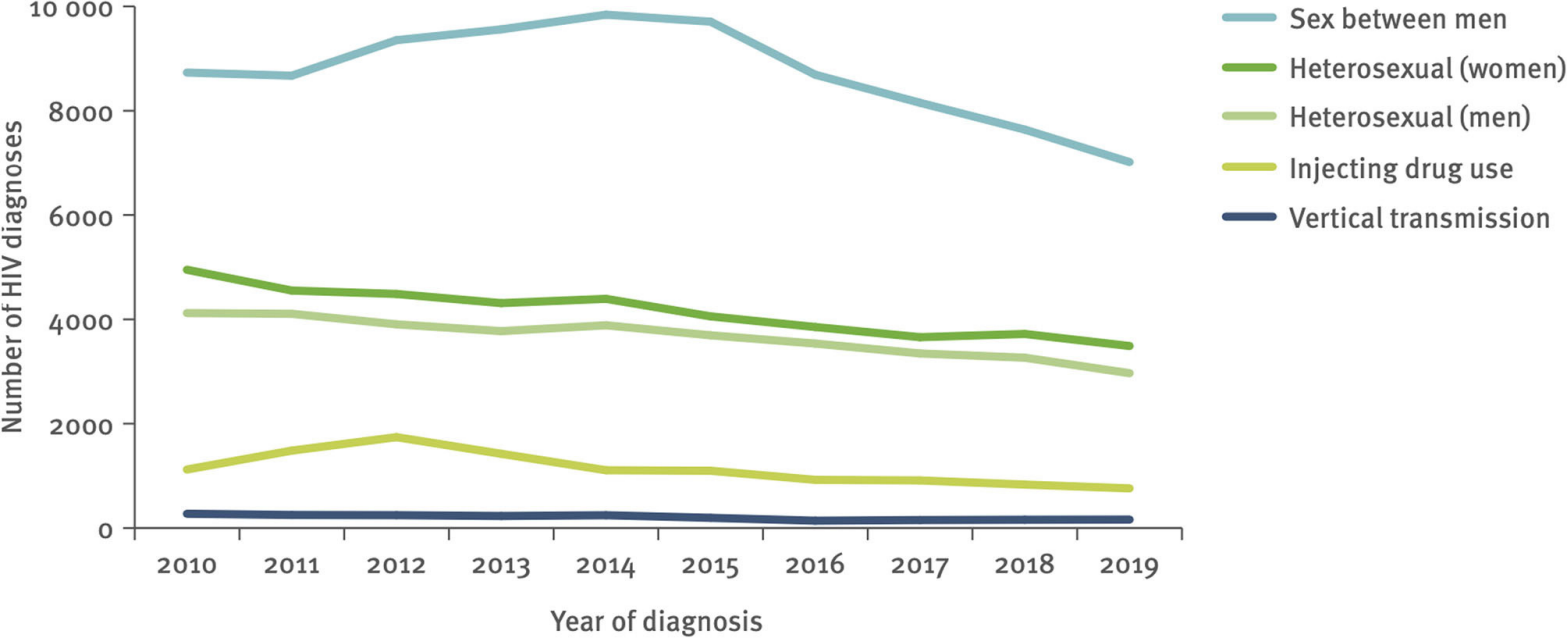
HIV diagnoses, by year of diagnosis and transmission mode, adjusted for reporting delay and missing transmission imputed, EU/EEA, 2010–2019. Retrieved from the ECDC/WHO HIV surveillance report for 2020 (16). Data from 24 EU/EEA countries included. HIV diagnoses reported by Iceland, Ireland, Malta, and Poland excluded due to incomplete reporting on transmission mode during some years of the period; diagnoses reported by Belgium, Italy, and Spain excluded due to incomplete reporting during a portion of the period.

Although a decline in the number of cases attributed to MSM is identifiable (Figure 1), until 2015, despite relatively high HIV treatment coverage and some well-established prevention programs with multiple interventions existing in most EU/EEA countries, the number of new HIV diagnoses had not decreased in this key population (17). The reasons for the high number of infections in these groups remain probably the same as before, being multifactorial and include elevated numbers of sexual partners among MSM, increased consumption of alcohol and recreational drugs during sex with one or more individuals, along with a reduction in consistent use of condoms for prevention (17).

In Europe, there is a need for reinforcement of available prevention tools that currently include: (i) health promotion, with information and education; (ii) consistent use of condoms; (iii) promotion of change in sexual behavior; (iv) regular testing for HIV infection; (v) antiretroviral therapy for the HIV-infected partner; and (vi) post-exposure prophylaxis with antiretrovirals with immediate start after at-risk sexual intercourse (18). It therefore seems irrefutable that the widespread implementation of a prevention tool such as PrEP could be of value for European MSM, “*as no HIV vaccine is yet available and male circumcision has not been shown to prevent HIV transmission via the anal route”* (18). There is still enough space for PrEP to be appropriately introduced and/or further developed into the national European HIV prevention and risk reduction strategies.

## Efficacy and Effectiveness of PrEP for MSM in the European Union

In the EU, introduction of PrEP was awaited with expectation. In some countries, like UK or the Netherlands, the annual numbers of HIV infections were still high at the time and not suggesting a significant decline among MSM (9). The main interventions used to prevent HIV-1 transmission in the EU included voluntary early testing programs, risk counseling, and the promotion of condom use (19). However, in view of the increasing number of new HIV infections worldwide, the range of prevention with screening, counseling, and condom needed further intensification (19). The first marketing authorization in the EU for PrEP came late in 2016, ~4 years later than in the USA, and considered by some “*a far greater gap than occurred in the rollout of antiretroviral therapy”* (17).

In the EU, any product related to prevention or treatment of HIV infection must follow the centralized procedure for marketing authorization. This means that any company developing a medicine intended to treat or prevent HIV infection needs to apply to European Medicines Agency (EMA) and go through this procedure. If this procedure is successful, the European Commission will grant a marketing authorization valid in all 27 Member States. Pricing and reimbursement are afterwards defined by each Member State. In July 2016, EMA recommended to the European Commission granting marketing authorization in the EU for emtricitabine/tenofovir disoproxil to be used for PrEP “*in combination with safer sex practices to reduce the risk of sexually acquired HIV-1 infection in adults at high risk”* (19).

Following the USA Food and Drug Administration (FDA) approval in 2012 of daily oral TDF/FTC for PrEP to prevent HIV infection in high-risk individuals in the USA, there was a sustained debate about implementing such prophylactic regimen in other geographic regions, including in Europe (18). Some questions frequently asked are why there was such debate at the time and why the need to show the relevance of existing data to the European population for approval of the first PrEP regimen in the EU. At the time, available studies were in fact mainly conducted in the African population, and the fact that such relevance was not produced did not deter the Committee of Human Medicinal Products (CHMP) of the EMA, since the committee was convinced of a positive benefit/risk as mentioned below.

As briefly mentioned before, the clinical trials performed until 2012 were randomized controlled trials of TDF/FTC or TDF alone. The five large phase III efficacy trials of oral PrEP with TDF or TDF/FTC conducted until 2012 and one other in 2015 (Table 1) in high-risk individuals led however to some differing results (18).

**Table 1:** Efficacy and adherence rates across PrEP trials (Partners PrEP, TDF2 Study, Bangkok TDF, iPrEx, FEM-PrEP and VOICE).

**Randomized trials (Authors)**	**Populations**	***n***	**Efficacy outcome (medicinal product)**	**Lower limit of CI**	Adherence^*^
Partners PrEP (20)	HC (Kenya, Uganda)	4,758	67% TDF; 75% TDF/FTC	44% TDF; 55% TDF/FTC	82%
TDF2 study (21)	YM&W (Botswana)	1,219	62% TDF/FTC	21.5%	80%
Bangkok TDF (22)	IVDU (Thailand)	2,413	49% TDF	9.6%	67%
iPrEx (23)	MSM (S. America, S. Africa, Thailand, USA)	2,499	44% TDF/FTC	15%	51%
FEM-PrEP (24)	YW (Kenya, South Africa, Tanzania)	2,120.	6% TDF/FTC	−52%	37%
VOICE study (25)	YW (South Africa, Uganda, Zimbabwe)	5,029	−49% TDF; −4% TDF/FTC	−130% TDF;−50% TDF/FTC	30%

*Based on data compiled by Molina et al. (18).*

*FTC, emtricitabine; TDF, tenofovir disoproxil fumarate; IVDU, intravenous drug users; MSM, men who have sex with men; HC, heterosexual couple; YM, young men; YW, young women; PrEP, pre-exposure prophylaxis; CI, confidence interval (95).*

* *Adherence was assessed by the proportion of participants with drugs detectable in plasma and who remained free of infection in the active PrEP arms*.

All trials mentioned had a similar design and aimed to evaluate the benefit of daily oral PrEP on the incidence of HIV (18). The efficacy outcomes (Table 1) range from 75% reduction in the incidence of HIV infection among serodiscordant couples (in the case of the Partners PrEP study) to a non-statistically significant 49% increase in the HIV infection incidence in the Vaginal and Oral Interventions to Control the Epidemic (VOICE) study in high-risk young women (18).

From the trials described in Table 1, only the “*Chemoprophylaxis for HIV Prevention in Men who have Sex with Men (iPrEX)”* study was conducted in MSM. In this trial, with individuals enrolled from low- and middle-income South American countries, the efficacy outcome was approximately 44% with a lower bound of the 95% confidence interval at only 15% (23). These values appear to be below the 30% efficacy target pre-defined in advance (23). This 30% value is not determined randomly. These 30% represent the lowest level for a public health benefit for a certain preventive intervention for most regulatory authorities (18, 26), but these interpretations date from 2009 and these days one may consider that there are other factors taken into consideration for a final regulatory decision. These values under 30% were not seen in other randomized clinical trials that, for example, established a 60% reduction of HIV infection incidence because of male circumcision, which led to implementing this intervention in many countries with high endemic HIV rates (18).

There has been some controversy regarding the reasons that could justify the differences in efficacy outcomes observed between PrEP trials (as reported in Table 1), with different explanations from different authors being proposed. These factors range from:

i) adherence to a daily regimen (18);
ii) unreliability of measuring of adherence by self-report or pill count compared to measure adherence via blood drug measurements (18);
iii) differences in gender, age, route of HIV acquisition, and rate of concomitant sexually transmitted infections among participants (18).

Many believe that these first results regarding PrEP efficacy were not very convincing, and therefore, the European Medicines Agency deferred a positive opinion on the marketing authorization for oral PrEP until further evidence was gathered, including the expected results from two European trials among MSM and transgender women: the PROUD study (conducted in England/UK) and the IPERGAY study (conducted in France and Canada) (27, 28). Although the results were already available, EMA ended up basing the approval of FTC/TDF in the EU mainly in the results of the iPrEX study (held in 6 countries around the world and none in the EU) and in the results of the Partners PrEP trial (recruiting from Kenya and Uganda) (20, 23).

The PROUD (Pre-exposure Prophylaxis to Prevent the Acquisition of HIV-1 Infection) study was an open-label efficacy trial that randomized 544 MSM accessing services at 13 British public sexual health clinics to receive PrEP at study enrollment (*n* = 275) or to a “*wait list”* control group (*n* = 269) where individuals received other HIV prevention services that included counseling, condoms, post-exposure prophylaxis, and sexually transmitted infection (STI) “*diagnosis and treatment but did not receive PrEP until after efficacy was demonstrated in the immediate treatment group”* (29, 30). As summarized on Table 2, an intent-to-treat (ITT) analysis (the relative reduction in HIV incidence among those assigned to receive active medication compared with placebo) could show that individuals assigned to the immediate PrEP group had an 86% reduction in HIV infections, compared to individuals in the “*wait list”* control group (29). As mentioned by Riddell et al. this represents “*3 HIV infections in the immediate PrEP group vs. 20 in the control group”* (30). The study could also show high adherence to daily use of PrEP and no significant occurrence of risk compensation (e.g., an increase in sexual risk behavior) among the MSM who took part (9).

**Table 2:** Randomized efficacy trials of oral TDF or TDF/FTC combination therapy for pre-exposure prophylaxis–IPERGAY and PROUD.

**Randomized trials (Authors)**	**Populations (Countries)**	***n***	**Efficacy outcome (study medicine)**	**Lower limit of CI**	Adherence^*^
IPERGAY study (27)	MSM (France and Canada)	400	86% TDF/FTC	39%	86%
PROUD study (28)	MSM (England–UK)	545	86% TDF/FTC	58%	100%

*Based on data compiled by Riddell et al. (30); originally captured from Mayer and Ramjee (31).*

*FTC, emtricitabine; TDF, tenofovir disoproxil fumarate; MSM, men who have sex with men; PrEP, pre-exposure prophylaxis; CI, confidence interval (95).*

* *Adherence was assessed by the proportion of participants with drugs detectable in blood samples and who remained free of infection in the active PrEP arms*.

The IPERGAY (Intervention Préventive de l'Exposition aux Risques avec et pour les Gays) study was conducted in France and Canada in MSM, randomizing individuals to Molina et al. (27), Riddell et al. (30):

a) receive pericoital TDF/FTC (two pills in between 2 and 24 h prior to anal intercourse and one pill daily for 2 consecutive days after sexual intercourse, not exceeding over seven pills in a week), or;
b) matched placebo for PrEP.

In comparison to the PROUD study (where PrEP was offered as continuous treatment to participants in the treatment group), there is a clear difference in the regimen offered in the IPERGAY study, where PrEP was used by participants “on-demand” or as needed (a regimen further discussed ahead and designated as event-driven PrEP or, in short, ED-PrEP), before risk exposure would happen and for another 2 days following the event (27, 30).

In this study, the ITT analysis (Table 2) revealed a reduction of 86% in HIV infections. As a result, “*when participants in the placebo group were offered open-label TDF/FTC, the efficacy increased to 97%”* (30). This study was the first to inform on the relevance of PrEP around sexual contact and showed the relevance of the regimen that includes the start of FTC/TDF in between 2 and 24 h prior to sex and continues for 2 days after last sexual intercourse every 24 h since the last pill (9), compared to placebo.

Even with several questions being raised on the generalizability of the results stemming from the IPERGAY study (e.g., participants in this study had on average at least one episode of unprotected sex per week and were highly adherent to the proposed regimen) (30), the French Government allowed early availability at a national level of this ED-PrEP regimen, as an alternative to the established daily use, using an existing legal alternative to marketing authorization. As mentioned above, this regimen showed a similar protection of 86% (9, 27).

Both PROUD and IPERGAY studies were developed based on a “process of consultation with the community starting with informed HIV treatment and prevention advocates who recruited other HIV and lesbian, gay, bisexual, and transgender sector advocates” (17). The influence of the community organizations in all this process is of special importance. In fact, community organizations had responsibility in providing support and counseling in the IPERGAY study, with both IPERGAY study and PROUD study having representatives of the community on the steering committees and community engaging strategies (17). These interested communities started with “patient and non-patient advocates of HIV treatment and prevention who recruited other HIV and lesbian, gay, bisexual, and transgender sector advocates” (17).

General awareness and interest in PrEP remained residual in the MSM community in Europe until 2014, but when asked about PrEP, several MSM expressed some interest in it (17). Back in 2012 the EMA had already made public a reflection on the non-clinical and clinical development of oral and topical HIV PrEP (32) enumerating the challenges and unsettled research questions, in agreement to what was already highlighted by the British HIV Association and the British Association for Sexual Health (33) and later discussed by Molina et al. in 2013 (17, 18).

Notwithstanding the considerations above, both EU based trials—the PROUD study and the IPERGAY study—confirmed the high protective effect of FTC/TDF used for PrEP (approximately 86% in both trials, as mentioned above) (9), and this is of special importance given the EU centric basis of both studies. With the demonstration of the efficacy of daily PrEP and event-driven PrEP to prevent HIV infection among MSM, in order to have a real impact in Europe, PrEP had to be taken and used appropriately by those at high risk for HIV infection and who will benefit the most (9).

Although initially the European Center for Disease Prevention and Control (ECDC) was more skeptical regarding PrEP (17), following the publication of the results of the PROUD study and IPERGAY studies in 2015, the ECDC finally stated that EU countries should consider the integration of PrEP into their existing HIV prevention programs for those at high risk for HIV infection, and this recommendation was also followed by the WHO (6).

This would only be possible if the EMA approved TDF/FTC as a fixed dose PrEP regimen to be used in all EU Member States. Gilead Sciences (the marketing authorization holder of Truvada®–the commercial name of TDF/FTC fixed dose combination—in USA and EU) needed to start the dialogue with the EU regulator to submit an extension of the existing treatment therapeutic indication to include the use of Truvada® as PrEP, similarly like the dialogue started by Gilead Science with the French Authorities following the publication of results of the IPERGAY study. Only after a positive opinion of the CHMP of the EMA, the European Commission would consider granting a marketing authorization valid in all Member States of the EU. Only after this important regulatory step, reimbursement and access may be considered in these different countries.

Considering the main studies supporting the marketing authorization of this extension of indication in the EU, “*the iPrEx study showed that FTC/TDF reduced the risk of HIV infection by 42% in HIV-negative men or transgender women who have sex with men and who were considered at high risk of HIV infection”* (19). The study compared FTC/TDF with placebo in 2,499 subjects who showed high-risk behavior such as inconsistent or no condom use during sexual intercourse (19). In the Partners PrEP trial study, conducted in 4,758 heterosexual serodiscordant couples, the same combination (vs. placebo) reduced the risk of becoming infected by 75% in the heterosexual partners of HIV-positive men and women (19). Both studies “*reinforced that the better the adherence to daily treatment with FTC/TDF the better the protection against HIV-1 infection”* (19). At the time EMA made this assessment, data were reported from the pilot phase of PROUD in MSM and was also taken into consideration as supportive data.

Still, although finally adopting a positive opinion and recommendation to the European Commission to grant this extension of therapeutic indication of TDF/FTC to include prophylaxis, when discussing the uncertainty in the knowledge about the beneficial effects, the CHMP clearly stated that: “*There are two issues that are expected to impact on the benefit of once daily Truvada in routine use over longer periods than have been studied within formal clinical trial settings. The first is the potential for dwindling adherence to daily dosing, which has already been shown very clearly to impact on efficacy. The second is that taking an oral PrEP will prompt at least some individuals to engage in more risky behaviors, which could result in a higher rate of seroconversion despite PrEP compared to the trial settings, especially if also accompanied by dwindling adherence. The most relevant investigation of these risks within a clinical trial setting was in the open label PROUD study. However, in this study that was specifically intended to mimic routine use, a proportion of subjects in the delayed group gained access to PrEP, anyway. Therefore, it is difficult to interpret the overall findings but, despite some access to PrEP in the delayed group, a larger proportion allocated to PrEP reported unprotected receptive anal intercourse (21 vs. 12%; p* = *0.03, test for trend). Several studies found that those engaging in unprotected receptive anal intercourse were more likely to be adherent and derived high levels of protection despite this behavior”* (34).

Despite these uncertainties, the EU regulator agreed that the degree of protection granted by TDF/FTC “*has been repeatedly shown to be related to the level of adherence, supported by finding drug in plasma and/or intracellularly, although the minimum concentrations that are needed to provide protection have not been identified”* (34). A marketing authorization for PrEP was formally approved in EU countries in the Summer of 2016.

## The First Post-Approval Steps and the Need to Clarify the Public Health Benefits of PrEP in Europe

The success of media coverage and targeted campaigns in the USA and Australia regarding the use of PrEP (35, 36) in order to raise awareness among MSM did not entirely hit Europe at the same level in the beginning. It was only in 2015, just before the EMA approved the use of TDF/FTC, that a visible wave of support for PrEP hit Europe, including several Pan-European campaigns and social media groups (such as the *Nous Sommes PrEP* group in France) (17).

Cairns et al. (17) further report that the benefit of using PrEP was considered “*to be modest, the costs to largely centrally funded health systems were substantial, and the model for delivery that would ensure adequate access was not clear”* (17). Transatlantic data originating from the USA contributed to this European skepticism. Although some studies showed that if ~20% of all MSM were to use PrEP in the USA, over 62,000 new cases of HIV infection could be prevented, resulting in a 10% decline of HIV infections at 20 years compared with no PrEP use (18, 37), the incremental cost of the healthcare budget would be significant, making PrEP very difficult to be considered a cost-effective strategy (18). Notwithstanding, by restricting the use of PrEP to individuals at high risk (defined in this case as MSM with over five sexual partners per year), approximately 41,000 cases of HIV infection would be prevented, with a comparable reduction of HIV prevalence by 10% at 20 years (18).

This further supported the need for effectiveness data generated in the EU to clarify the benefits of PrEP as a public health tool and to optimize both access and models of delivery. In a “Letter to the editor” of the International Journal of STD & AIDS, dated and published in 2015, Kenyon and Osbak proposed to find out how many MSM in Europe could benefit from PrEP and called it “the 9 billion Euros question” (38). If the 2014 USA Centers for Disease Control and Prevention (CDC) and WHO guidelines were followed, PrEP should be prescribed to adult MSM “*who are HIV-negative, have had a male sexual partner in the past 6 months, are not in a mutually monogamous relationship with a recently tested HIV-negative man and at least one of the following:*

a) *any anal condomless intercourse (receptive or insertive) in the past 6 months;*
b) *any STIs diagnosed or reported in the past 6 months;*
c) *is in an ongoing sexual relationship with an HIV-positive male partner”* (38).

According to the conditions of this study, approximately 1.4 million MSM in the EU would qualify for PrEP, from the estimated population of 5 million MSM aged 18–64 years old (38). The price of a year's supply of commercially available standard PrEP in the EU is estimated to be ~6,500 Euros or 8,100 US$ (38). The same authors conclude that “*at this price, daily doses would cost 9.1 billion Euros per year for the 1.4 million men in the EU, excluding the other costs associated with PrEP implementation, which would require substantial health service infrastructure and staffing, and community education for MSM”* (38).

When analyzing available scarce data at the time, Cairns and colleagues suggest that, in the UK, when trying to make PrEP cost-effective for a larger group of individuals at risk, price cuts of 50–80% in PrEP would need to be enforced (17). For PrEP to be cost-effective in Europe, high-risk groups needed to be targeted. This could very well be considered a major deterrent of the widespread use of PrEP in Europe and, most probably, elsewhere.

Between 2015 and 2017 the practical applicability of PrEP as a complement to the current HIV prevention strategy in Belgium was studied, and no new infections were detected in the group of 200 gay men taking FTC/TDF as prevention. Since mid-2017, this treatment has been approved for reimbursement in Belgium following the marketing authorization granted by the European Commission (39).

In 2019, Hoornenborg et al. reported the results of the AMPrEP study, concluding that “*although the overall incidence of STIs did not change during 2 years of PrEP use, the incidence of STIs was higher among participants using daily PrEP than those using event-driven PrEP, which is likely a result of differences in sexual behavior*” (40). Another study showed that ED-PrEP could be a satisfactory alternative to daily PrEP for MSM who are at high risk, including periods of less frequent sexual intercourse (41), allowing individuals to adapt the uptake of PrEP according to any changes occurring in their sexual lives (41). The results were instrumental in the WHO decision to update the guidelines (as detailed further ahead) (42). It is clear that there is a need to tailor prevention interventions according to behavioral profiles, and a need to consider this dimension in the overall impact of access to PrEP in any country. At the same time, there is growing interest in developing better versions of available combinations, and an example is the introduction of tenofovir alafenamide (TAF) to substitute TDF, since the combination with TAF is non-inferior to the TDF/FTC therapeutic or prophylactic regimen leading to a more favorable bone density and renal biomarker profile (43).

Worth mentioning is also the role of post-exposure prophylaxis (PEP) not to be confused with PrEP. In the case of PEP, antiretrovirals are administered after exposure to prevent acquiring HIV and this is based on the fact that HIV may take up to 72 h to be detected in lymph nodes and up to 5 days to be detected in blood post-exposure (9). If antiretroviral drugs are administered within this “window of opportunity,” virus replication might be stopped, hence preventing the development of an infection (9). The current recommendation for PEP remains to take the fixed dose combination of three antiretrovirals (same as in treatment) starting within those 72 h post-exposure and prolonging for 28 days (9).

## Implementation and Access to PrEP as Part of a Combination Prevention Strategy for MSM in Europe

Sex between MSM remains the predominant mode of HIV infection transmission reported in Europe, accounting for half of all new HIV infection diagnoses where the transmission route is known (6, 15). While this is acknowledged, ~500,000 MSM in the EU (who would be very likely to use PrEP) cannot access it. But this is not much different from what happened in the USA. Although PrEP was firstly authorized in the USA in 2012, only about 10% of those individuals that might be expected to benefit from this intervention have started medication de facto (44). With MSM, there is evidence supporting an association between the willingness of MSM to use PrEP and an increased risk for sexually transmitted HIV (6, 45).

There is no implementation without first raising awareness. So, despite the approval of the marketing authorization for PrEP with TDF/FTC in the EU by the European Commission after a positive opinion of the EMA, individual Member States still had to decide whether to formally adopt PrEP as a public health tool and under which type of reimbursement scheme (e.g., formal reimbursement via governmental health budget or informal reimbursement, such as special projects or schemes). During this period, it was of vital importance to understand the level of awareness, knowledge, and predisposition to PrEP use within populations of interest in these countries.

So far, even in 2021, the differences between countries regarding raising awareness on PrEP are still very clear, with the UK, Netherlands, Belgium, and France leading the group with visible and notorious campaigns, with less polarized public positions, dialogues, and campaigns compared to the USA (17). This is most probably related not only to the early experience in these countries, hosting relevant clinical trials such as PROUD and IPERGAY, but also with other studies started in 2015 such as the Amsterdam PrEP Study (AMPrEP) (40) and the Be-PrEP-ared Project (held in Antwerp, Belgium) (39), both studies aiming for the collection of real-world data on the uptake of PrEP among MSM at high risk for HIV infection (9).

In Germany, in 2015, from a sample of 20 volunteers (mean age 35.9 years old, and regarding HIV status, 35% were HIV-positive and 65% where HIV-negative), all participants were aware of the existence of PrEP (albeit not having been marketed yet in Germany) and were also knowledgeable of the existence of PEP which was already considered in national guidelines (46). The same study could show a general favorable attitude toward PrEP and also a high demand for such intervention, with several individuals describing schemes to gain access to PrEP (e.g., via another country where it was authorized, active search within the community or even actively distributing from home, as described by one participant) (46). Although most findings in this study may be considered anecdotal, they do clearly point out that MSM in Berlin were prepared to accept and take PrEP as soon as it would become available (46).

In Spain, in 2016, in a sample of 866 volunteer MSM recruited over the internet or at HIV testing centers, 28.7% were aware of the existence of PrEP and 57.6% confirmed they would use it if it was available (47). In the same study, 16.6% of the volunteers said they would be unwilling to use PrEP and 25.8% were unsure (47). Other important information was gathered based on this study, namely, that men who had already heard of PrEP were more willing to use and had more favorable opinions regarding PrEP, and that the favorite providers for PrEP were doctors (91%) and pharmacists (83.3%) (47).

Also, in Spain, using an online survey and taking advantage of the realization of the World Gay Pride 2017 in Madrid, Iniesta et al. were determined to test “*the awareness, knowledge, use, and willingness to use and need of PrEP among MSM and transgender women (TW)”* who attended the event (48). This study could show that among the 472 MSM attending the World Gay Pride 2017, there was little awareness of PrEP, low accuracy of PrEP knowledge, but a significant need and willingness to use PrEP (48).

In 2017, Goedel et al. looked into awareness of PEP among MSM in London, using a sample of MSM “using a geosocial-networking smartphone application” (an “app”) (49). These apps, such as “*Grindr”* (https://www.grindr.com) currently represent the most common virtual context platform for MSM to meet their sexual partners (49, 50), with a Press Release from the company reporting seven years ago having over two million daily users in over 200 different countries and in London the highest number of users in the world (49). It is acknowledged that “*MSM who use these, or similar apps may often engage in high-risk behaviors where PEP use may be a suitable prevention strategy”* (49). In this London-based study, most individuals of a sample of 179 MSN reported having heard of PEP (88.3%) and 27.4% reported having used it (49). The same authors showed that knowledge of PEP existence was associated with “*the disclosure of one's sexual orientation to their general practitioner and reporting one's HIV status as negative (rather than unknown)”* (49). The study showed that individuals reporting recent use of recreational club drugs were more associated with having used PEP (49).

It is undeniable that the ability to use TDF/FTC post-exposure could be a valuable risk-reduction approach (in addition or in the absence of PrEP) that deserves better attention in the EU. We have already mentioned the results of a demonstration study in Amsterdam, the AMPrEP project (51), and also here authors showed that a significant number of study participants had a clear preference for daily use of PrEP instead of an event-driven use. This majority of participants preferring daily use of PrEP presented with a high number of condomless anal sex episodes before the initiation of a PrEP regimen, with a high prevalence of STIs (51). This study identified that at least in this European population, the determinants of event-driven PrEP or PEP were (51):

i) older age;
ii) less situations of condomless anal sex episodes;
iii) not taking any other daily medications, and;
iv) being involved in a stable relationship.

The National Fund for Health Research in the UK funded a study able to provide initial information and tendencies for PrEP use and initiation among MSM who are HIV-negative, using available data from a prospective cohort (that recruited MSM who were HIV negative or of unknown HIV status from two large sexual health clinics in London and one in Brighton) while the roll-out of PrEP in England was being planned (52). In England, in the period between 2013 and 2018, even with access to PrEP only via the IMPACT trial, both awareness and use of this preventive tool by MSM increased noticeably during this time (52). The authors of this study conclude that an improvement of access to PrEP by routine appointment by the National Health Service England could translate into a significant “*increase in PrEP use among all eligible MSM but should include public health strategies to target socioeconomic and demographic disparities in knowledge and use of PrEP”* (52).

Taking everything into account, we may agree that after the IPERGAY study results came out, intermittent PrEP use (before and after sexual intercourse) could be an effective and cheaper approach compared to daily (uninterrupted) PrEP (27, 38). So, one aspect that needs to be properly addressed from a public health perspective is that there may be an optimal price for PrEP to be negotiated with governments/reimbursement authorities and insurance companies, depending on particular populations at different levels of risk (38). The two major determinants of the cost-effectiveness of this intervention appear to be the price of the drug used for PrEP and HIV incidence (28). Even without a clear update or change on the guidance from WHO and USA CDC, the European AIDS Clinical Society recommended this ED-PrEP regimen for MSM, reducing of the amounts of drug required for daily administration and reducing the costs with the drug in about a half (28, 53). In 2019, following the results of the demonstration studies available, WHO recognized the need to consider event-driven (ED) PrEP as an additional option for MSM and updated the recommendations (42). The WHO followed other authors and mostly based their change of recommendation on the results of the interim analysis of the ANRS PREVENIR Study (41). Situations when ED-PrEP could be considered a valid alternative to daily PrEP, according to the WHO, are detailed in Table 3. Of significant note, although this alternative regimen to daily PrEP may be considered by WHO and other guidelines, this posology was not assessed by EMA, and therefore, from a regulatory perspective, this corresponds to an off-label use of this medicine.

**Table 3:** Situations when event-driven (ED) PrEP could be considered.

**For whom is ED-PrEP appropriate**	**For whom is ED-PrEP NOT appropriate**
• A man who has sex with another man: - Who would find ED-PrEP more effective and convenient; - Who has infrequent sex (e.g., sex <2 times per week on average); - Who is able to plan for sex at least 2 h in advance, or who can delay sex for at least 2 h	• Cisgender woman or transgender woman; • Transgender man having vaginal/frontal sex; • Man having vaginal or anal sex with woman; • People with chronic hepatitis B infection

*Adapted from the update to WHO's recommendation on oral PrEP in 2019 (42)*.

**Figure 2:**
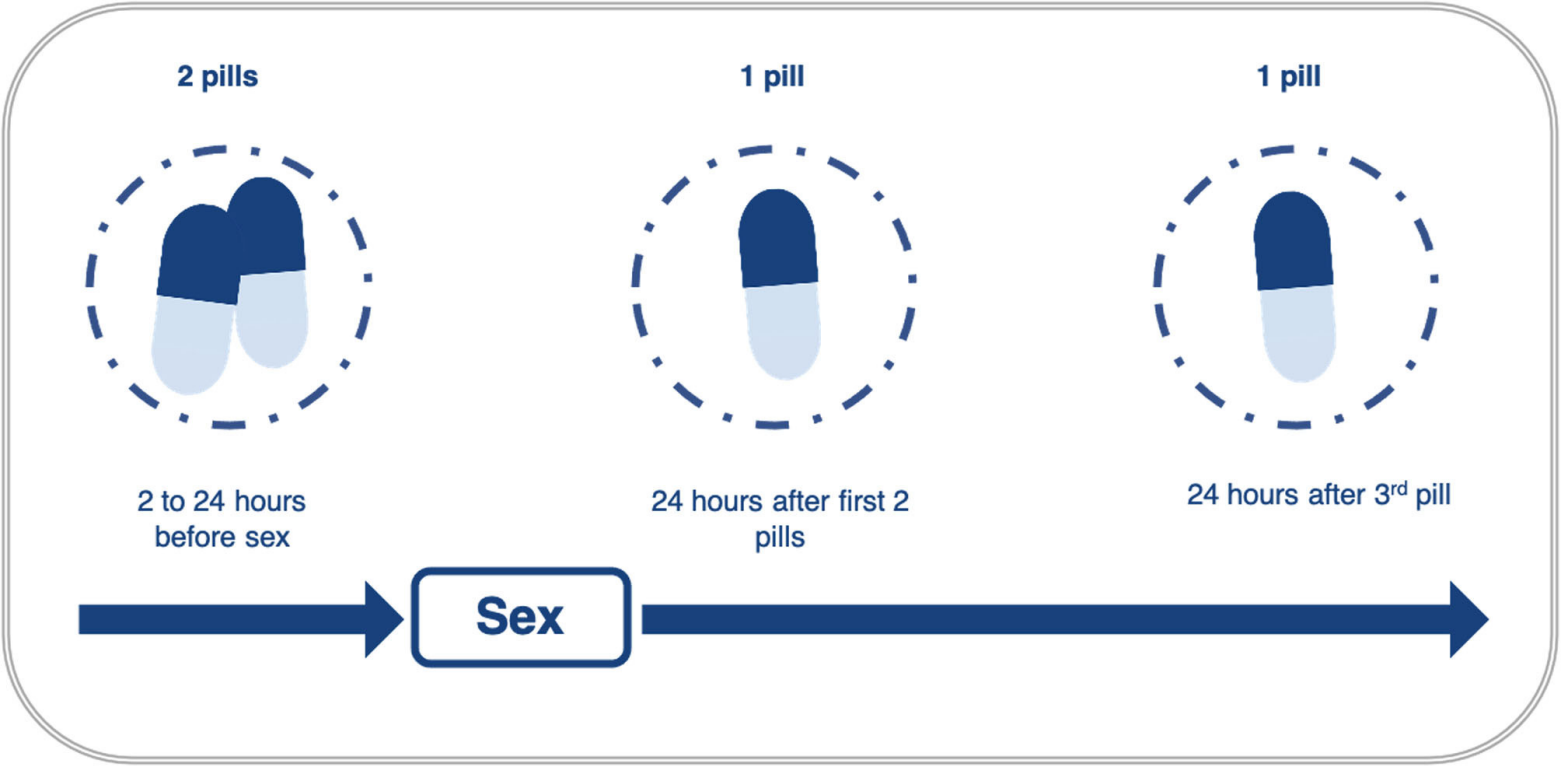
Schematic representation of the ED-PrEP 2 + 1 + 1 regimen, adapted from the update to WHO's recommendation on oral PrEP in 2019 (42).

As previously mentioned for the clinical demonstration trial, ED-PrEP for MSM starts with the administration of a loading dose comprising two pills of TDF/FTC between 2 and 24 h before sexual intercourse, followed by a third pill 24 h after the first two pills, and by a fourth pill 48 h after (Figure 2), on the 2 + 1 + 1 rule (42). If sexual intercourse continues beyond 1 day, MSM using ED-PrEP can stay protected by taking another pill each day as long as sex continues and stopping 2 days after the last sex act as per the initial 2 + 1 + 1 rule (42).

The WHO is very cautious regarding the use of ED-PrEP in adolescent MSM below 18 years old, since no clinical trials have been conducted in this population (42). There remains an ample consensus around the fact that daily PrEP works better than non-daily PrEP in adolescent MSM (below 18 years old), as per the results of the ADAPT/HPTN 067 study (42). There is currently insufficient evidence supporting ED-PrEP in populations at risk other than MSM; therefore, it is recommended that women, transgender women, and men who have vaginal and/or anal sex with women are prescribed another PrEP regimen (42).

Three years following the marketing authorization for PrEP in Europe, some scholars, the medical community and the LGBTQ+ community questioned the implementation of this strategy at least in the EU area, since all shared the common concept that the longer the delay in access to PrEP for this population, the more HIV infections will occur (6). Until now, information publicly available regarding the access, implementation, and uptake of the strategy in Europe is scarce. The only available overview of the current situation was published in October 2019 (6). Other than this review, available information was compiled and shared by advocacy groups (such as PrEP in Europe, www.prepineurope.org) and patient associations.

Other than the demonstration or implementation studies conducted in Europe (e.g., England, Netherlands, and France), Germany prepared a scheme (in 2017) to give access to people who wish or need PrEP at affordable prices from doctors and pharmacies who adhered to the scheme (54).

According to “PrEP in Europe” (55), the other way people in Europe are having access to PrEP is online, buying it from wherever it is offered in the World Wide Web. Although in some countries online pharmacies may offer generic versions of TDF/FTC in the same fixed-dose combination found in Truvada® at a cheaper price, this is not the reality in all the EU (especially because until the marketing recommendation by the EMA it would not even be legal), and online buying (outside certified pharmacies) comes sometimes at the higher cost of people buying counterfeit low quality products that have no traceable origin and are a Public Health matter of concern.

Advocacy groups in the EU, such as the group “I Want PrEP Now” and “PrEPster” have publicized PrEP purchase and similar groups all around Europe have done the same (54). According to the same source, ~130,000 people in the USA were taking PrEP in 2017, out of the 1.2 million likely candidates (54). In Europe, just under 3,000 people in France were receiving PrEP in 2017 via the healthcare system and up to 150 in Norway (54). Around 10,000 people in Europe were purchasing PrEP for personal use, so possibly over 10,000–15,000 people in Europe could already take PrEP in 2017 at their own expense (54).

Hayes et al. published an analysis of the implementation and access to PrEP in Europe and Central Asia in 2019 (6). This work reflects information collected between January and March 2019. The immediate information stemming out of this study is that in Europe there is substantial diversity regarding the implementation of PrEP among EU Member States (6).

**Figure 3:**
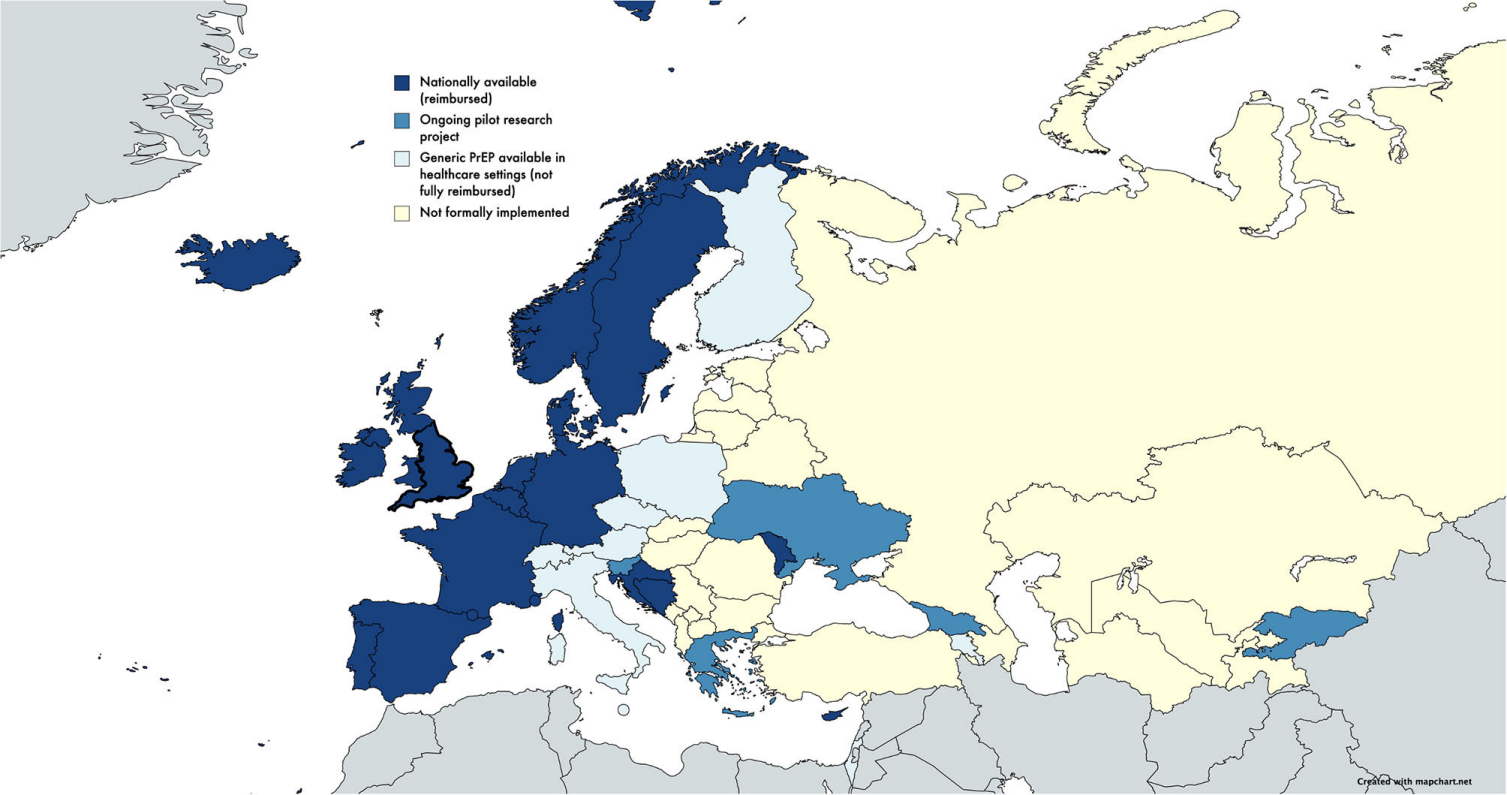
Status of PrEP implementation in Europe and Central Asia by October 2020 based on data reported by ECDC in the implementation, standards, and monitoring operational guidance; created using mapchart.net (8).

More recently, the ECDC further recognized the discrepancy in the scale-up of PrEP implementation across the EU/EEA and UK, and an update (reflecting available information on the 20th of October 2020) was provided (Figure 3) (8). In 2020, in the EU/EEA and UK, the evolution in access (nationally available and reimbursed) is noted for Ireland, England, Wales, and Spain, in comparison to the data reported for 2019, while ongoing pilot projects remain in some European countries. But, still, areas without any formal implementation of PrEP dominate Eastern Europe and parts of Euroasia, as seen in Figure 3 (note: this Figure is based on data as reported by ECDC, and the word “reimbursed” should be interpreted in the broader sense of formal and informal reimbursement schemes).

With these identified disparities, the aim of the ECDC was to facilitate the development of guidance that would support countries in their attempts to implement PrEP in Europe (8). A guidance document was recently published by ECDC, providing an overview of key markers of preparedness to deliver larger-scale PrEP programs, engaging different stakeholders and helping to prioritize PrEP within national health agendas (8).

When considering the barriers previously reported in Europe regarding PrEP, the most commonly cited barrier was the cost of the drug (6). In 2021, ~6 years after marketing authorization was granted in Europe, it is very clear that PrEP is not reaching the entire population at high risk as it should. According to Hayes et al. other barriers identified include (6):

i) limited technical capacity to consider PrEP;
ii) the cost associated to service delivery;
iii) feasibility;
iv) concerns raised regarding increased transmission of other STIs;
v) concerns about a reduction in condom consistent use;
vi) adherence to PrEP;
vii) the development of drug resistances, and;
viii) beliefs that there is no clearly identified group with sufficiently high incidence in accordance with the WHO guidelines (6).

In order to mitigate the existing barriers and promote PrEP implementation across Europe, minimum standards on the principles of establishing PrEP programs, monitoring, and surveillance need to be agreed upon “*and include guidance on identifying and estimating the size of the key populations in need of this intervention, which can then inform program targets”* (6). It is expected that health authorities in these different countries, especially in countries that are part of the EU, channel their efforts to the improvement of the accessibility to PrEP not only for MSM but also for both women and heterosexual men at high risk for HIV (6). It is however acknowledged, and based on the experience gained in the USA, that although “*protocols to identify individuals are most likely to benefit from PrEP have been developed, addressing racial, ethnic, and socioeconomic disparities continue to pose additional challenges”* (44).

The issues around the price and cost of PrEP could be resolved if special encouragement is given to manufacturers to produce and invest in these drugs, given the loss of market protection that occurred in 2017 for Truvada® allowing for the generic market to flourish (28). If the issues around the cost of PrEP (either daily or ED-PrEP) become resolved or secondary, there will still be additional issues to be addressed by Member States to implement prevention strategies that comprise different approaches and include PrEP (28).

**Figure 4:**
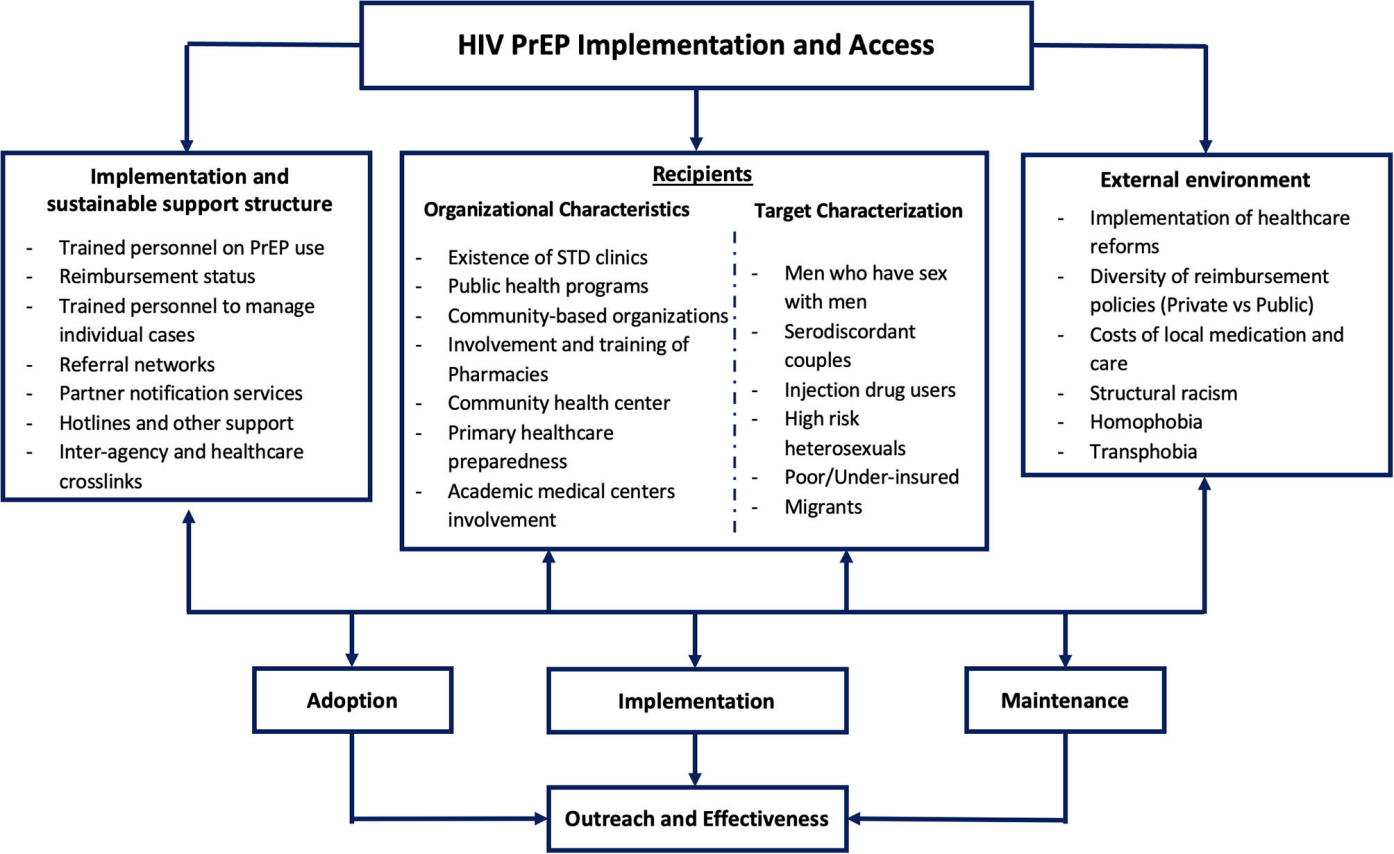
Factors involved in PrEP implementation and access as originally proposed by Mayer et al. (44), with adaptations.

Making use of implementation science, EU Member States will have to continue to consider different strategies that might be useful in the adoption of PrEP among health organizations considering the unique organizational barriers and facilitators that each one may have for a sustainable delivery (44). Mayer et al. (44) based on the USA experience, proposed an ecosocial model of factors involved in PrEP implementation (Figure 4).

It is undeniable that best practices for optimizing PrEP delivery based on clinical practice, outreach tailored programs, and evidence are still needed (2). The optimization of PrEP scale-up is challenging, but many believe this is the only way forward (44). Combining PrEP with treatment scale-up in San Francisco (USA), London (UK), and New South Wales (Australia) led to substantial reductions in new HIV infections (56). The use of ED-PrEP also opens new opportunities for optimization of PrEP use across Europe.

In terms of the way services are organized in the EU, services provided in the region are variable ranging from open access and/or free services ensuring testing and treatment for HIV and STIs, to situations where access is only possible via significant co-payments or even extreme situations of “*limited access to non-confidential and pejorative services”* (28). In the EU, like most countries, most of the budget for healthcare comes from public funding (through collection of taxes and social insurance contributions) (28). There might be however a small contribution (<5%) from private schemes (28). In some EU countries (such as the Netherlands, Germany, Portugal, etc.), healthcare is delivered by both public and private sectors but funded also through insurance schemes and/or formal and informal copayments (28). Independent of the level of public or private services provided, the importance, significance, and determination of community-based organizations who frequently organize to offer HIV and STIs screenings, adjusted to key populations of interest, are undeniable (28). These services end up collaborating with primary healthcare services for PEP and the prescription of antiretrovirals (28).

From many factors or barriers discussed before, that undermine the full implementation of PrEP in Europe, affordable access appears to be one of the first issues that need urgent intervention. Mechanisms to support not only the costs of medication (either reimbursement by health authorities or insurance) but also the costs of care need to be studied. Stigma should not be an issue for people that want to have access to PrEP since many private insurance companies have been associating access to PrEP to sexual risk behaviors that could affect how the health plans of these people are negotiated.

According to a recent editorial in *The Lancet HIV*, “*the funding of PrEP in England is a sorry saga”* (56), further elaborating that in 2016, the National Health Service (NHS) England decided that HIV prevention fell within the remit of local public health authorities, and subsequently, NHS was not responsible for funding (56). Subsequent legal challenges via court actions led to a court ruling that NHS England could fund PrEP, and in September 2018, the drug patent extension for Truvada® was overturned by a High Court in England and generic cheaper forms of FTC/TDF became available (56). The same authors also state that “*although it is now clear that funding should no longer be an obstacle to universal access, to date there seems to be no resolution among health authorities for how to fund long-term PrEP roll-out in England”* (56).

European countries need to focus their long-term plans for HIV infection on scaling up preventive services. Given the overwhelming evidence available to support the effectiveness of PrEP, not pursuing this path is a public health missed opportunity. The need for high coverage; fair cost; reimbursement schemes; rapid roll-out; and consideration of the health, social, and geographical inequalities faced by many of the individuals at risk for HIV will be needed to maximize the preventive effects of PrEP (56) not only in Europe but in many other areas of the globe. For the greatest impact in Europe, PrEP should be truly available, and access should be given to all who need it. In the words of McCormack back in 2016, “*the momentum to implement PrEP in European countries is increasing and provides a welcome opportunity to expand and improve clinical services and civil society support focused on HIV and related infections including other sexually transmitted and blood-borne infections”* (28). In fact, the momentum was there, but it still leaves much to achieve.

Experts seem to agree that we need to overcome not only barriers inherent to health care systems but also other societal barriers that restrict access to high-quality care (4). Several initiatives are helping to provide proof of the principle that the elimination of HIV infections, stigma, discrimination, and deaths are all workable (4). Projects such as the “Getting to Zero” initiative in San Francisco serve as models “*for implementation of a combination of treatment as prevention and PrEP at the local, regional, national, and global levels”* (4).

At the program level it continues to be important that condoms keep their central role, as it is widely agreed that there is no intention for PrEP to replace condom use, even if taken as prescribed (9). It was the consistent use of condoms that prevented millions of infections among MSM around the world. Some authors went one step further and even defined possible ways of conveying the message about the combination use of PrEP and condoms (9):

a) PrEP is not meant to replace the use of condoms but, “*if taken as prescribed, PrEP on its own has the same high level of protection against HIV as consistent condom use”* (9);
b) the combination of PrEP with condom use provides not only safest protection against HIV (9); and
c) the consistent use of condoms, if viable and suitable, provides a high level of protection for both HIV and STIs, and in such cases, PrEP may not be necessary (9).

PrEP and condoms should continue to be combined as a strategy, especially when PrEP remains costly in some EU countries (despite the existence of generics) and condoms have the advantage of also protecting from other STIs (9).

One of the issues that were considered a barrier to PrEP implementation in the EU was precisely the possible association to the increase of STIs when (or if) PrEP was implemented. There had been a historic low number of STI cases reported during the years when there were not so many therapeutic options available for HIV. The increase in the number of STIs after the year 2000 is inevitable and parallel to the first significant therapeutic advancements in the management of HIV, when the fears of the consequences of contracting HIV reduced (57). With the “*awareness of the efficacy of PrEP and treatment as prevention”* as a measure to control HIV in the populations at risk (including MSM), the rise in STIs sped up since 2013 (57).

Based on the demonstration study AMPrEP, Hoornenborg et al. investigated if PrEP (either daily or ED-PrEP) could promote risk compensation, “defined as increased sexual risk behaviors” leading to higher incidence of STIs (40). Interestingly, the study found that although the overall incidence of STIs did not change significantly along the 2 years of PrEP use, the incidence of STIs was higher among individuals who preferred ED-PrEP, this being most likely related to differences in sexual behaviors adopted by these participants (40).

The findings of the above study are aligned with the findings from a recent study by Jansen et al. that investigated the prevalence of chlamydia, gonorrhea, and mycoplasma in MSM in Germany (58). The authors report a high prevalence of STIs among MSM (e.g., PrEP users) being asymptomatic (58). The findings of this study support once again that a significant proportion of PrEP users practice condomless sex and reinforces the need for low-threshold and free-of-cost counseling and thorough screening for STIs (58). One important aspect also raised in this study is the need to address the use of party or recreational drugs by PrEP users (58).

Behavioral change is one of the most tricky and challenging strategies in Public Health. There are some interventions that need to be taken into consideration at the same time PrEP is considered, making this a multicomponent intervention. There is still plenty of room for improvement for strategies that have a clear focus on health promotion, behavioral change, and HIV prevention, and these should include PrEP and PEP, especially in particularly vulnerable populations within MSM (as the challenging example of chemsex). Targeted interventions would be clearly beneficial, with the potential for only being needed for short periods of time while translating into longer-term benefits in terms of HIV prevention and STIs (if associated to condom use) (59). A study conducted in MSM living in Paris (France) confirmed that rectal douching is a common practice mostly associated with condomless sexual intercourse, with participation in group sex, with HIV infection, STI diagnosis, and likelihood to use rectal microbicide gels (60).

Douching can breakdown the protective rectal epithelium, thereby increasing susceptibility to HIV and other STIs (61), therefore PrEP (including ED-PrEP) and condoms could have an important role for individuals (especially receptive or “passive” partners) who feel more comfortable douching before sexual intercourse and do not want to be at increased risk.

A behavior common among MSM relates to the significant proportion of these individuals who use inhaled nitrites, or poppers, to enhance sexual intercourse. A survey was conducted in 2016 in 580 MSM living in Paris (France) regarding the use of poppers, condomless sexual anal intercourse, serosorting, sexual positioning, use of PrEP, PrEP candidacy and even interest in different possibilities for PrEP delivery (62). The study showed that popper users were more likely to consider themselves suitable candidates for PrEP, while showing that they were most probably not current or past users of PrEP (62). Explanations for the belief that these individuals would be suitable candidates and actively considered PrEP were related to increased serosorting and condomless anal sexual intercourse reported by these participants (62). Also emerging from this survey is the enormous interest demonstrated in alternative PrEP delivery options, namely, long-acting injectable versions of PrEP (62).

Given the long demand for long-acting versions of PrEP, several companies have tried to develop versions of PrEP that would be attractive for those with issues related to adherence or who prefer a once-a-month administration, for example. In October 2020, the EMA recommended to the European Commission granting a marketing authorization to Rekambys® (active substance: rilpivirine) and Vocabria® (active substance: cabotegravir) to be used together for the treatment of HIV infection (63). The EU regulator based its opinion on data from phase III randomized, open-label, multicenter clinical trials including HIV-infected men and women above 18 years old and asymptomatic, who were either treatment naïve or where already under treatment of a standard of care (63). Although this is still for use in a treatment setting, the evidence available at the time is enough to support both efficacy and safety of a regimen including both drugs administered every 4 or 8 weeks (63). This seems in line with the expectations of some patients living with HIV, since the availability of a regimen including a long-acting antiretroviral allows the reduction of the dosing frequency and the burden associated with daily pill taking (63).

The importance of having drugs such as Rekambys® and Vocabria® approved for treatment is paving the way for the same approach to be studied, developed, and authorized for PrEP in Europe and worldwide in a very near future. Some trials are already ongoing and study HPTN 083 already showed the superiority of cabotegravir in comparison to TDF/FTC for the prevention of HIV (64).

## Conclusions: Where to go From Here?

It is now well-established that PrEP should be considered a significant additional prevention tool for MSM, although there is a tendency in Europe to still promote access only to those who are considered being at high risk, although more MSM would probably benefit from these interventions.

Applying the concept of HIV prevention cascades in the EU will most probably lead to a significant increase in coverage, mainly by targeting: (i) interventions on the demand-side (improving risk perception, awareness and uptake of prevention approaches; (ii) interventions on the supply side (prevention products, procedures, and health structures more available and accessible, and; (iii) adherence interventions (supporting ongoing adoption of prevention behaviors, including those not involving any of the prevention products) (65).

Successful implementation of PrEP needs a defined model of care appropriate to the size of the target population and capacity of the local health system (8), and it needs to be built on national commitments to address all identified structural, capacity, and policy barriers to PrEP implementation (8, 44). The ECDC recently identified key principles that should guide countries for effective PrEP implementation (8), including stakeholder engagement, creation of stigma-free environments, PrEP awareness (with demand creation), and the consequent update of clinical and public health guidelines with definition of standardized eligibility (promoting population wide access based on need criteria) and clear linkage to care reinforcing combination STI and HIV prevention (8).

Overall, special boost should be given to ED-PrEP as it might be more cost-effective, safe, and highly effective for MSM independently of assuming a passive (“receptive”) or active (“insertive”) role in the sexual intercourse (42). It is expected that countries update their treatment guidelines to include the option of ED-PrEP (42) alongside with the daily PrEP option, promoting, reinforcing, and supporting educational campaigns and dedicated care for the target population. Independent of the regimen or route of administration, PrEP represents a unique opportunity for engagement of health structures and professionals with individuals, on all issues surrounding their sexual health (42).

A recent publication from Bavinton and Grulich (66) clearly highlights the importance of contextualizing the new PrEP modalities that have emerged and are emerging, reinforcing the need to better understand the long-term patterns of PrEP use in different target populations and developing models of use by these individuals, alternating through “*periods of use and non-use, as well as switching between dosing regimens or modalities as they become available”* (66).

While COVID-19 has created an unprecedented crisis around the world, and although resources are most probably currently directed to fighting this global threat, the fight against HIV should never stop being a priority. Ending HIV must remain in the political, societal and health agendas around the world and we, members of modern societies, should not have any doubts about who is at risk for infection of HIV and to whom scaling up of PrEP is a crucial step. Better use of the already available tools is in order, PrEP is one of the most promising interventions Europe should aim for given the intrinsic potential for impacting the HIV epidemic (66).

## Author Contributions

BS drafted the manuscript. All authors provided substantial input in the study design, provided a critical revision of the manuscript, read, and approved the final manuscript.

## Conflict of Interest

The authors declare that the research was conducted in the absence of any commercial or financial relationships that could be construed as a potential conflict of interest.

## Publisher's Note

All claims expressed in this article are solely those of the authors and do not necessarily represent those of their affiliated organizations, or those of the publisher, the editors and the reviewers. Any product that may be evaluated in this article, or claim that may be made by its manufacturer, is not guaranteed or endorsed by the publisher.
